# Benign or aggressive? Understanding spinal melanocytomas in comparison to malignant melanoma

**DOI:** 10.1007/s11060-025-05350-0

**Published:** 2025-12-01

**Authors:** Alice Ryba, Theresa Mohme, Johannes Kerschbaumer, Emily von Bronewski, Vanessa Hubertus, Vicki M. Butenschoen, Andreas Kramer, Felix C. Stengel, Martin N. Stienen, Marco Battistelli, Filippo Maria Polli, Hanno S. Meyer, Lasse Dührsen, Klaus Christian Mende, Manfred Westphal, Florian Ringel, Bernhard Meyer, Julia Onken, Claudius Thome, Sven O. Eicker, Malte Mohme

**Affiliations:** 1 Department of Neurosurgery, Medical Center Hamburg-Eppendorf, Hamburg, Germany; 2 Wirbelwerk Hamburg, Hamburg, Germany; 3 Department of Neurosurgery, Medical University of Innsbruck, Innsbruck, Austria; 4 Department of Neurosurgery, Charité University Hospital Berlin, Berlin, Germany; 5 Department of Neurosurgery, School of Medicine, Technical University Munich, Munich, Germany; 6 Department of Neurosurgery, University Medical Center Mainz, Mainz, Germany; 7 Department of Neurosurgery and Interdisciplinary Spine Center, H-OCH Health Ostschweiz, St. Gallen, Switzerland; 8 Department of Neurosurgery, Fondazione Policlinico Universitario “A. Gemelli” IRCCS, Rome, Italy; 9 Friedrich Ebert Hospital, Neumünster, Schleswig-Holstein Germany; 10 Department of Spine and Scoliosis Surgery, Lubinus Clinicum, Kiel, Germany; 11 Laboratory for Brain Tumor Biology, Department of Neurosurgery, University Medical Center Hamburg-Eppendorf, Martinistr. 52, 20253 Hamburg, Germany

**Keywords:** Spinal melanocytoma, Meningeal melanocytoma, Meningeal melanoma, Melanocytic tumor

## Abstract

**Introduction:**

Spinal melanocytic tumors are rare, with limited data on their clinical course and aggressiveness. Since intraoperative dark pigmentation and infiltrative margins can lead to misclassification of MC as MM—especially in the absence of a known primary—this multicenter study characterizes melanocytomas (MC) and contrasts them with malignant melanoma (MM).

**Methods:**

We retrospectively analyzed 56 patients with spinal intradural melanocytic tumors (2010–2024) from seven European neurosurgical centers. Clinical, radiological, histological, and surgical features were analyzed. Univariate and multivariate analyses were performed to identify prognostic risk factors.

**Results:**

The study included 22 patients with spinal MC and 34 patients with MM. Median age was comparable (61 vs. 58 years, *p* = 0.09), but MC patients had a longer symptom history (13 vs. 1.3 months, *p* = 0.0001) and more often intramedullary tumors (72.7% vs. 2.9%, *p* < 0.0001). Gross total resection was achieved in 40.9% of MC cases and 61.8% of MM cases (*p* = 0.07), with instrumentation required more often for MM (*p* = 0.0022). Ki-67 proliferation index was significantly higher in MM than MC (26.7% vs. 6.6%, *p* < 0.0001). Postoperative deficits were more frequent in MC (54.6% vs. 14.7%, *p* = 0.002) and MCs showed higher frequency of ataxia postoperatively (36.4% vs. 11.8%, *p* = 0.04). MM patients received more adjuvant therapy (88.2% vs. 31.8%, *p* = 0.0001), with a trend toward higher local recurrence (26.5% vs. 14.7%, *p* = 0.31).

**Conclusion:**

Our study demonstrates that MC, though considered benign, carries a high risk of postoperative deficits. Furthermore, GTR was achieved in only 40% of cases, underscoring its locally infiltrative nature and potential for semi-benign biology.

**Supplementary Information:**

The online version contains supplementary material available at 10.1007/s11060-025-05350-0.

## Introduction

Meningeal melanocytomas (MC) are rare benign primary melanocytic lesions of the CNS with an incidence of approximately 1 case per 10 million people annually [1–4]. They arise from leptomeningeal melanocytes, which are predominantly located along the ventrolateral medulla oblongata and upper cervical spinal cord. Therefore, they are most commonly found in the posterior fossa, although they can also be found in the spinal region. According to the 2021 World Health Organization (WHO) classification, primary melanocytic tumors of the central nervous system (PMN-CNS) include four distinct entities: meningeal melanocytosis, meningeal melanomatosis, MC, and primary meningeal (malignant) melanoma [5, 6]. Although MC are historically considered benign lesions, they may display intermediate or uncertain malignant potential, including local recurrence and clinically aggressive courses despite resection. In recognition of this clinical variability, Brat et al. proposed a histopathological grading system that further stratified these tumors into low-grade MC, intermediate-grade variants, and malignant melanoma, based on criteria such as mitotic activity, nuclear atypia, and proliferative index [2]. In contrast, MC have a high-grade, aggressive counterpart—malignant melanoma (MM) of the spine, arising either as a primary malignant melanoma (pMM) or as malignant melanoma metastases (mMM). Although MCs may share overlapping radiological and histological characteristics with MMs, including melanin pigmentation, intradural localization, or leptomeningeal spread, MMs are defined by distinct malignant behaviors, including rapid growth, extensive infiltration of surrounding CNS tissue, elevated mitotic rates, and poor clinical outcomes [4, 7].

The macroscopic appearance can also mislead intraoperative assessment: intense black pigmentation and occasional infiltration may prompt a provisional diagnosis of MM, especially when no primary melanoma is evident [8, 9]. Moreover, melanomas can remain clinically occult, the primary may be atypically located, asymptomatic, or may even regress, so several CNS melanoma series report metastases without an identifiable cutaneous primary [10–12]. Although differentiating between MC and primary MM of the spine (or even non-CNS-derived metastatic malignant melanoma) remains challenging, it is essential to determine the appropriate therapeutic strategy and estimate prognosis. In addition, owing to their rare occurrence and the limited literature with only case reports available, no definitive gold standard therapy has been established for spinal MC. However, complete resection remains the primary therapeutic goal whenever possible [13].

The aim of the present study was to systematically compare spinal MC and MM (including both primary and metastatic melanoma) in a multicenter cohort, with a particular focus on identifying clinical, radiological, surgical, and histopathological predictors of benign versus malignant behavior. Additionally, we aimed to evaluate whether our surgical treatment strategies should differ in relation to the biological profile of the tumor and how these factors may inform subsequent individualized therapeutic approaches.

## Methods

This retrospective multicenter study analyzed 56 patients with intradural, intra- or extramedullary spinal melanocytic tumors between 2010 and 2024. Data were collected from seven European tertiary neurosurgical centers. Data analysis was performed retrospectively on anonymized datasets. This multicenter retrospective analysis was conducted in accordance with the ethical principles of the Declaration of Helsinki. The study was based exclusively on anonymized datasets derived from routine clinical diagnostics and follow-up obtained within standard patient care at the participating neurosurgical centers. No additional analyses—histological, molecular, or questionnaire-based—were performed beyond clinical routine. The clinical, radiological, histological, and surgical features were analyzed. Univariate and multivariate analyses were performed to identify prognostic risk factors.

### Patients’ characteristics

Patient characteristics included age at diagnosis, sex, tumor type, preoperative symptoms, postoperative complications, and clinical outcomes at follow-up. The duration of symptoms (in months) before the first surgical intervention was recorded. Neurological status was assessed both pre- and postoperatively based on the presence of pain, motor deficits, sensory disturbances, vegetative dysfunction (bowel/bladder), and ataxia. Functional status was evaluated using the modified McCormick scale and documented preoperatively, at hospital discharge, and at the last follow-up.

Postoperative outcomes were classified into three categories: unchanged, improved, or worsened, both at discharge and at the final follow-up visit. Recorded postoperative complications were categorized using standardized definitions and included hemorrhage, cerebrospinal fluid (CSF) fistula, infection, and cardiovascular events.

Adjuvant treatment following surgery was categorized as no further treatment (follow-up imaging only), radiotherapy (RTx), chemotherapy (CTx), combined radiochemotherapy (RTx + CTx), or repeat surgery reflected treatment decisions recorded in institutional tumor boards. Long-term follow-up parameters included the duration of follow-up in months, McCormick grade at the last follow-up, and the occurrence and duration of local and distant tumor recurrence. Death and the corresponding date of death were recorded, whenever applicable.

### Radiographic parameters

Preoperative magnetic resonance imaging (MRI) was used to assess the tumor volume within the spinal canal; the extent of canal occupation was classified into four categories: <25%, 25–50%, 50–75%, and > 75% of the cross-sectional area of the spinal canal. Additionally, we evaluated intramedullary tumor location, dural involvement, presence of extraspinal extension, spinal level localization, and number of involved vertebral segments. Tumor localization was categorized into the craniocervical, cervical, thoracic, lumbar, and sacral regions.

### Surgical parameters

All the patients underwent microsurgical treatment. Intradural procedures included approaches via the interlaminar window, hemilaminectomy, laminectomy, or laminoplasty with or without spinal stabilization. The extent of resection (EOR) was classified according to uniform radiographic and operative criteria (GTR = no visible residual tumor; STR = < 10% residual volume on postoperative MRI; debulking = > 10% residual tumor). Use of intraoperative neuromonitoring (IONM) was also recorded.

### Histopathological parameters

Diagnoses were established locally by board-certified neuropathologists at the participating tertiary centers using harmonized criteria based on the WHO 2021 classification of primary melanocytic neoplasms of the CNS. Tumors were classified, based on histopathological evaluation, as either MC or MM. The MM group comprised both primary and metastatic MM involving the spinal leptomeninges. Additionally, the Ki-67 proliferation index (%), S100 expression status, and presence of BRAF mutation were documented.

### Data harmonization and bias mitigation

Given the retrospective multicenter design, potential heterogeneity was minimized through a standardized case report template with uniform definitions for all key variables across centers. To reduce referral and selection bias, all participating institutions were high-volume tertiary neurosurgical centers with comparable surgical indications and access to adjuvant treatment modalities. Data entry and cleaning were centralized at the coordinating center, and any ambiguous or missing values were cross-checked with local investigators before inclusion in the final analysis.

### Statistical analysis

Statistical analyses were performed using GraphPad Prism version 9. Figures were generated using Adobe Illustrator. Continuous variables were analyzed using an unpaired two-tailed Student’s t-test, and categorical comparisons between two groups were assessed using two-sided Fisher’s exact test. Given the exploratory nature of this study and the limited sample size inherent to ultra-rare tumors, no formal correction for multiple comparisons was applied. Statistical significance was set at *p* < 0.05.

## Results

**Fig. 1:**
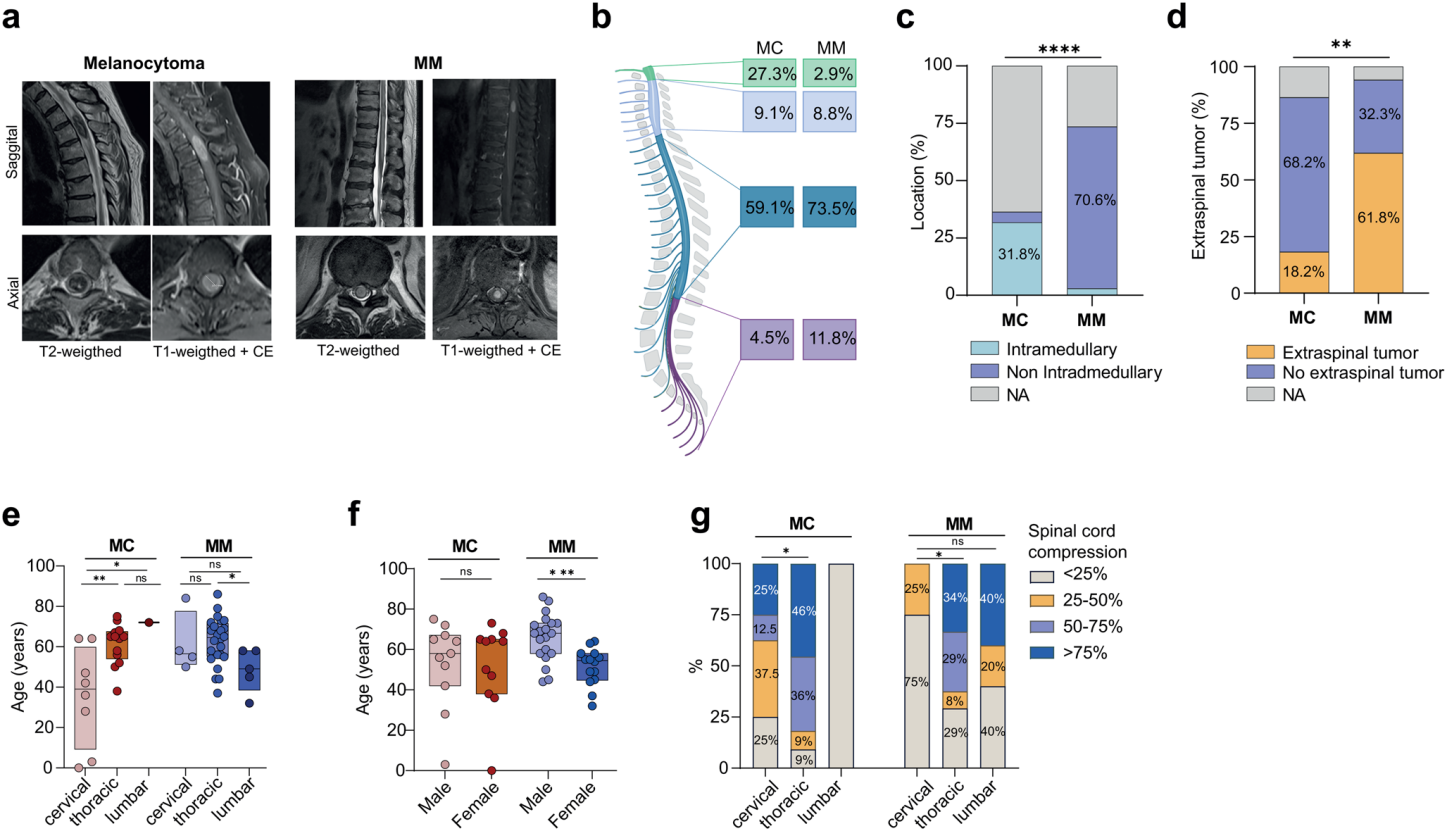
Radiological and demographic characteristics of spinal melanocytoma (MC) and malignant melanoma (MM). **a**) Preoperative MR Imaging of one MC patient with an intramedullary lesion in T2-T3 level of the spine and one MM patient. Both show a hypointense lesion in T2-weighted imaging and hyperintensity in T1-weighted imaging with contrast enhancement (CE). **b**) Tumor localization along the spine differed significantly, with craniocervical involvement more common in MC (27.3% vs. 2.9%, *p* = 0.0117). **c**) Intramedullary location was significantly more frequent in MC compared to MM (31.8% vs. 2.9%, *p* < 0.0001). **d**) MM cases were more likely to present with extraspinal tumor components (61.8% vs. 18.2%, *p* = 0.0034). **e**) Age distribution by tumor location, showing relevant differences across segments, with melanocytomas showing a significantly younger mean age when located in the cervical spine compared to thoracic or lumbar localization **f**) Age distribution stratified by sex and diagnosis revealed that males with MM were significantly older than females, whereas no sex-related age difference was observed in the MC group. **g**) Distribution of spinal cord compression by location and entity, showing similar compression patterns across both groups with significantly more compression in the thoracic spine

A total of 56 patients were included, comprising 22 (39.3%) with spinal MC and 34 (60.7%) with MM. An overview of the patient characteristics and a comparative analysis between MC and MM are provided in Table 1. To rule out major confounders within the malignant melanoma cohort, we compared primary CNS melanomas (pMM) and metastatic melanomas (mMM) (Supplementary Table 1). Primary lesions were exceedingly rare, accounting for only 20.6% (7/34) of all MM cases. We did not observe relevant differences in baseline characteristics or progression-free survival (PFS) between pMM and mMM (median 2.8 vs. 7.1, *p* = 0.24). However, pMM were associated with a significantly shorter overall survival (OS) compared with mMM (median 1.2 vs. 8.9, *p* = 0.02; HR 32.8, 95% CI 1.7–641.8). Patients with MC had a median age of 61 years (range 0.25–75) and a balanced sex distribution with a female-to-male ratio of 1:1, similar to patients with MM, who had a median age of 58 years (range, 32–86) and a male-to-female ratio of 1.4:1. On MRI, both entities frequently exhibited intrinsic T1 hyperintensity and T2 hypointensity, resulting in substantial radiological overlap and underscoring the difficulty of reliably distinguishing MC from MM based on conventional imaging alone (Fig. 1a). The craniocervical region was the most common tumor location in MC, occurring significantly more frequently than in MM (27.3% vs. 2.9%, *p* = 0.0117; Fig. 1b). In contrast, cervical, thoracic, and lumbosacral localization was observed at similar frequencies in both groups, without statistically significant differences. In addition, intramedullary tumor growth was significantly more frequent in the MC group (31.8% vs. 2.9%; *p* < 0.0001; Fig. 1c). There were no statistically significant differences between MC and MM in terms of tumor volume, number of involved spinal segments or dural involvement. However, additional extraspinal tumor manifestations were more frequently observed in MM (61.8% vs. 18.2%; *p* = 0.0034; Fig. 1d). Furthermore, tumor location was found to vary with age in melanocytomas, such that MCs located in the cervical spine presented a significantly younger mean age than thoracic or lumbar lesions (Fig. 1e). Additionally, sex-specific age differences were observed in MM, with males in the MM group being significantly older than their female counterparts (Fig. 1f). The distribution of spinal cord compression according to tumor location showed similar overall patterns in both cohorts, with significantly more pronounced compression in the thoracic spine (Fig. 1g).

**Table 1:** Patient characteristics and comprehensive comparison between MC and MM

	Melanocytoma	Malignant melanoma	*p* value
Total - n (%)	22 (39.3)	34 (60.7)	
**Clinical**			
Age - median (range)	61 (0.25-75)	58 (32–86)	0.0977
Gender - n (%)			0.5880
Male	11/22 (50)	20/34 (58.8)	
Female	11/22 (50)	14/34 (41.2)	
**Radiographic**			
Volume MR imaging - n (%)			0.8519
< 25%	4/22 (18.2)	9/34 (26.5)	
< 50%	4/22 (18.2)	4/34 (11.8)	
< 75%	5/22 (22.7)	8/34 (23.5)	
> 75%	7/22 (31.8)	12/34 (35.3)	
n.a.	2/22 (9.1)	1/34 (2.9)	
Intramedullary location - n (%)	7/22 (31.8)	1/34 (2.9)	**< 0.0001**
Dural involvement - n (%)	9/22 (40.9)	17/34 (50)	0.7817
Extraspinal tumor - n (%)	4/22 (18.2)	21/34 (61.8)	**0.0034**
Whole spine MRI - n (%)	15/22 (68.2)	27/34 (79.4)	0.7285
Localization - n (%)			
craniocervical	6/22 (27.3)	1/34 (2.9)	**0.0117**
cervical	2/22 (9.1)	3/34 (8.8)	> 0.9999
thoracic	13/22 (59.1)	25/34 (73.5)	0.3800
lumbar/sacral	1/22 (4.5)	4/34 (11.8)	0.6381
n.a.	0/22 (0)	1/34 (2.9)	
No. of Segments - n (%)			0.5134
1	10/22 (45.5)	17/34 (50)	
2	7/22 (31.8)	7/34 (20.6)	
3	4/22 (18.2)	3/34 (8.8)	
> 4	1/22 (4.5)	4/34 (11.8)	
n.a.	0/22 (0)	3/34 (8.8)	
Further spinal manifestations - n (%)	2/22 (9.1)	12/34 (35.3)	0.0525
**Surgical**			
Resection - n (%)			0.0760
GTR	9/22 (40.9)	21/34 (61.8)	0.1725
STR	10/22 (45.5)	6/34 (17.7)	**0.0351**
Debulking	1/22 (4.5)	5/34 (14.7)	0.3862
Biopsy	2/22 (9.1)	1/34 (2.9)	0.5548
n.a.	0/22 (0)	1/34 (2.9)	
Procedure - n(%)			**0.0022**
Interlaminar fenestration	3/22 (13.6)	2/34 (5.9)	
Hemilaminectomy	6/22 (27.3)	5/34 (14.7)	
Laminectomy	5/22 (31.8)	7/34 (20.6)	
Lamioplasty	7/22 (31.8)	2/34 (5.9)	
Stabilization	0/22 (0)	17/34 (50)	
n.a.	1/22 (4.5)	1/22 (4.5)	
IONM - n (%)	14/22 (63.6)	14/34 (41.2)	0.1707
Postop complications - n (%)			
Bleeding	0/22 (0)	2/34 (5.9)	
CSF fistula	3/22 (13.6)	0/34 (0)	
Infection	1/22 (4.5)	0/34 (0)	
Cardiovascular	0/22 (0)	0/34 (0)	
Nothing	16/22 (72.7)	30/34 (88.2)	
**Histopathology**			
Ki 67 (%) - mean (SD)	6.66 ± 7.73	26.70 ± 17.68	**< 0.0001**
S100 Positive - n (%)	12/22 (54.4)	21/34 (61.8)	0.9534
BRAF Mutation - n (%)	1/22 (4.5)	10/34 (29.4)	0.1000
**Outcome**			
Duration of symptoms (months) - mean (SD)	13.05 ± 15.91	1.34 ± 2.23	**0.0001**
Preop symptoms /deficits - n (%)			
None	2/22 (9.1)	2/34 (5.9)	0.6416
Pain	10/22 (45.5)	17/34 (50)	0.7895
Motor	6/22 (27.3)	14/34 (41.2)	0.3942
Sensitive	13/22 (59.1)	11/34 (32.4)	0.0586
Vegetative	4/22 (18.2)	5/34 (14.7)	0.7266
Ataxia	7/22 (31.8)	7/34 (20.6)	0.3634
Preop McCormick - n (%)			0.7513
0	1/22 (4.5)	0/34 (0)	
1	8/22 (36.4)	11/34 (32.4)	
2	6/22 (27.3)	8/34 (23.5)	
3	6/22 (27.3)	10/34 (29.4)	
4	1/22 (4.5)	3/34 (8.8)	
5	0/22 (0)	1/34 (2.9)	
n.a.	0/22 (0)	1/34 (2.9)	
Preop McCormick - mean (SD)	1.9 ± 1.0	2.3 ± 1.1	0.2320
New deficit postop - n (%)	12/22 (54.6)	5/34 (14.7)	**0.0026**
Postop outcome - n (%)			**0.0012**
Better	5/22 (22.7)	22/34 (64.7)	
Same	8/22 (36.4)	10/34 (29.4)	
Worse	9/22 (40.9)	2/34 (5.9)	
Symptoms /deficits at discharge - n (%)			
None	4/22 (18.2)	8/34 (23.5)	0.7463
Pain	2/22 (9.1)	2/34 (5.9)	0.6416
Motor	10/22 (45.5)	16/34 (47.1)	> 0.9999
Sensitive	13/22 (59.1)	12/34 (35.3)	0.1029
Vegetative	5/22 (22.7)	3/34 (8.8)	0.2408
Ataxia	8/22 (36.4)	4/34 (11.8)	**0.0448**
McCormick at discharge - n (%)			0.1900
0	1/22 (4.5)	0/34 (0)	
1	7/22 (31.8)	12/34 (35.3)	
2	4/22 (18.2)	11/34 (32.4)	
3	6/22 (27.3)	8/34 (23.5)	
4	4/22 (18.2)	1/34 (2.9)	
5	0/22 (0)	2/34 (5.9)	
McCormick at discharge - mean (SD)	2.2 ± 1.2	2.1 ± 1.1	0.6560
CSF pathological cells - n (%)	0/22 (0)	0/34 (0)	
Cerebral metastases - n (%)	1/22 (4.5)	10/34 (29.4)	0.0264
Systemic metastases - n (%)	1/22 (4.5)	25/34 (73.5)	**< 0.0001**
Systemic therapy before 1st diagnosis - n (%)	0/22 (0)	21/34 (61.7)	**< 0.0001**
Former spine surgery - n (%)	2/22 (9.1)	4/34 (11.8)	0.7520
**Follow-Up**			
Therapy after surgery - n (%)			**0.0001**
None	15/22 (68.2)	4/34 (11.8)	
RTx	2/22 (9.1)	10/34 (29.4)	
CTx	1/22 (4.5)	5/34 (14.7)	
RTx + CTx	2/22 (9.1)	15/34 (44.1)	
Re-Surgery	1/22 (4.5)	0/34 (0)	
Re-Surgery + RTx	1/22 (4.5)	0/34 (0)	
Follow-up [months] - median (range)	36.3 ± 47.4	8.1 ± 9.8	**0.0072**
Clinical course FUP - n (%)			0.9390
Better	5/22 (22.7)	8/34 (23.5)	
Same	7/22 (31.8)	12/34 (35.3)	
Worse	6/22 (27.3)	8/34 (23.5)	
n.a.	4/22 (18.2)	6/34 (17.7)	
McCormick at last FUP - n (%)			0.4985
1	5/22 (22.7)	11/34 (32.3)	
2	5/22 (22.7)	6/34 (17.6)	
3	1/22 (4.5)	6/34 (17.6)	
4	3/22 (13.6)	4/34 (11.8)	
5	2/22 (9.1)	1/34 (2.9)	
NA	6/22 (27.3)	6/34 (17.6)	
McCormick at last FUP - mean (SD)	2.5 ± 1.5	2.2 ± 1.2	0.5554
Local recurrence (LR) - n (%)	3/22 (13.6)	9/34 (26.5)	0.3152
Distant recurrence (DR) - n (%)	2/22 (9.1)	5/34 (14.7)	0.4025
Death - n (%)	1/22 (4.5)	14/34 (41.2)	**0.0067**

**Fig. 2:**
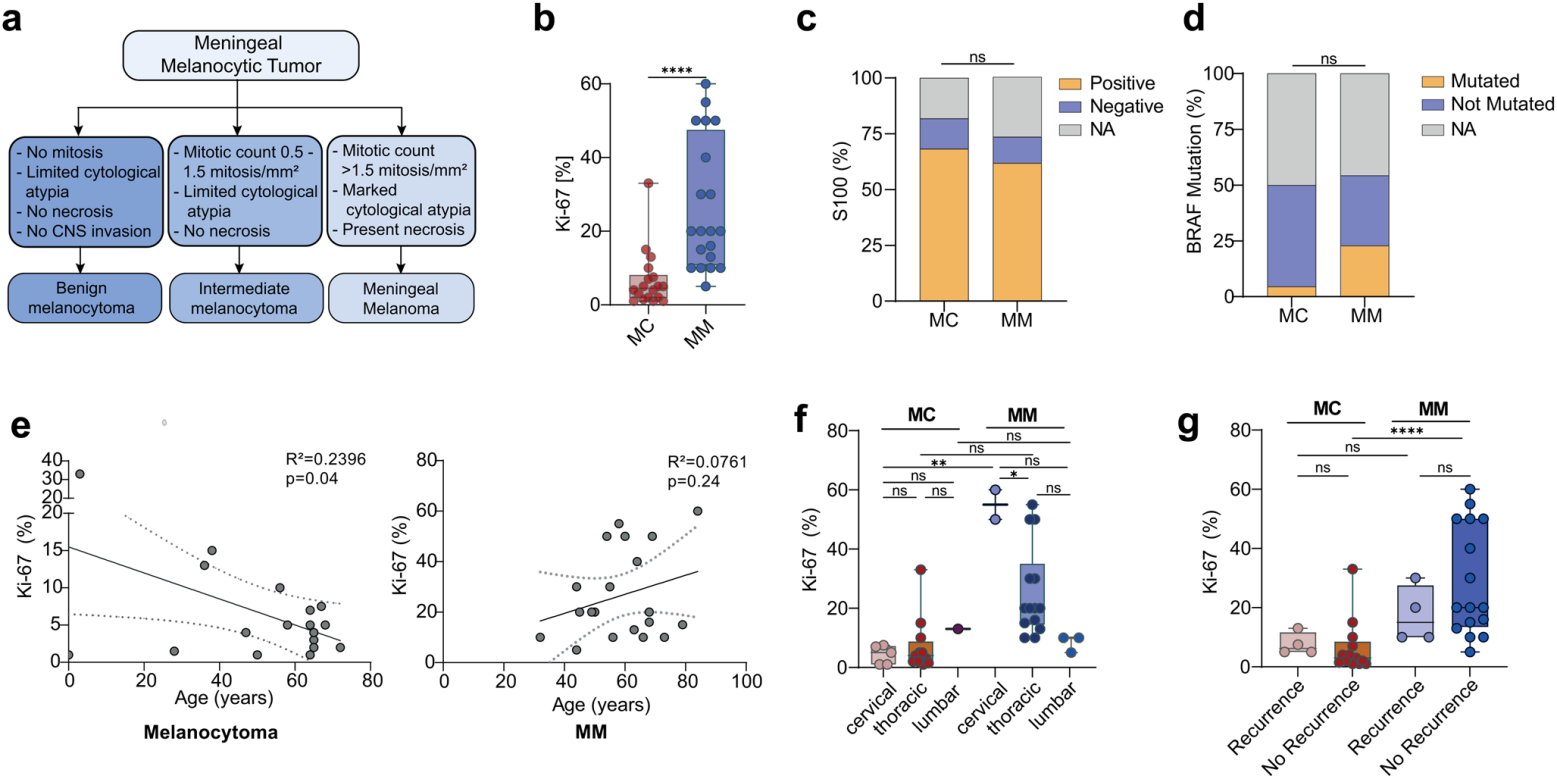
Histopathological profile of spinal melanocytic tumors. **a**) Classification framework of meningeal melanocytic tumors, ranging from historically benign melanocytoma to malignant melanoma., according to Brat et al. [2] **b**) Ki-67 proliferation index was significantly higher in MM than in MC (26.7% vs. 6.7%, *p* < 0.0001). **c**) S100 immunoreactivity was observed in the majority of both groups without significant differences (*p* = 0.9534). **d**) There were no statistically significant differences in the appearance of BRAF mutations (*p* = 0.1) **e**) Ki-67 correlated inversely with patient age in MC (R^2^ = 0.2396, *p* = 0.04) but not in MM (R^2^ = 0.0761, *p* = 0.24). **f**) Location-dependent variation in Ki-67: MM depicted significantly elevated indices when located in the cervical region (*p* = 0.0032). **g**) In MM, tumors without recurrence showed significantly higher Ki-67 levels compared to non-recurrent MCs (29.0 *±* 18.7 vs. 6.4 *±* 8.6, *p* < 0.0001)

According to the histopathological grading proposed by Brat et al., we included all cases of spinal MC (Fig. 2a) [2]. Histopathological analysis revealed a significantly higher Ki-67 proliferation index in MM than that in MC (26.7% vs. 6.7%, *p* < 0.0001; Fig. 2b). S100 expression was common to both groups and showed no statistically significant difference (61.8% vs. 54.4%, *p* = 0.9534; Fig. 2c). BRAF mutations were more commonly observed in MM patients, although the difference was not statistically significant (29.4% vs. 4.5%, *p* = 0.1; Fig. 2d). We further discovered that in the MC group, Ki-67 levels were inversely correlated with patient age (R² = 0.24, *p* = 0.04), while no such correlation was found in the MM group (R² = 0.08, *p* = 0.24; Fig. 2e). In addition, a location-dependent variation in Ki-67 expression was observed exclusively in MM, with significantly higher proliferation indices in cervical lesions (Fig. 2f). Furthermore, Ki-67 expression was significantly lower in non-recurrent MCs than in non-recurrent MM tumors (Fig. 2g).

Regarding the EOR, GTR was achieved in 40.9% and 61.8% of MC and MM cases, respectively (*p* = 0.076), whereas STR was more frequent in MC (45.5% vs. 17.7%, *p* = 0.0351). Stabilization procedures were required exclusively for patients with MM (50% vs. 0%; *p* = 0.0022). The use of IONM was comparable between the two cohorts (41.2% vs. 63.6%, *p* = 0.1707). Postoperative complications were rare in both groups. CSF fistulas occurred in three MC patients (13.6%) and no MM patients (0%), whereas minor bleeding complications were noted in two patients with MM (5.9%) and no MC patients (0%).

**Fig. 3:**
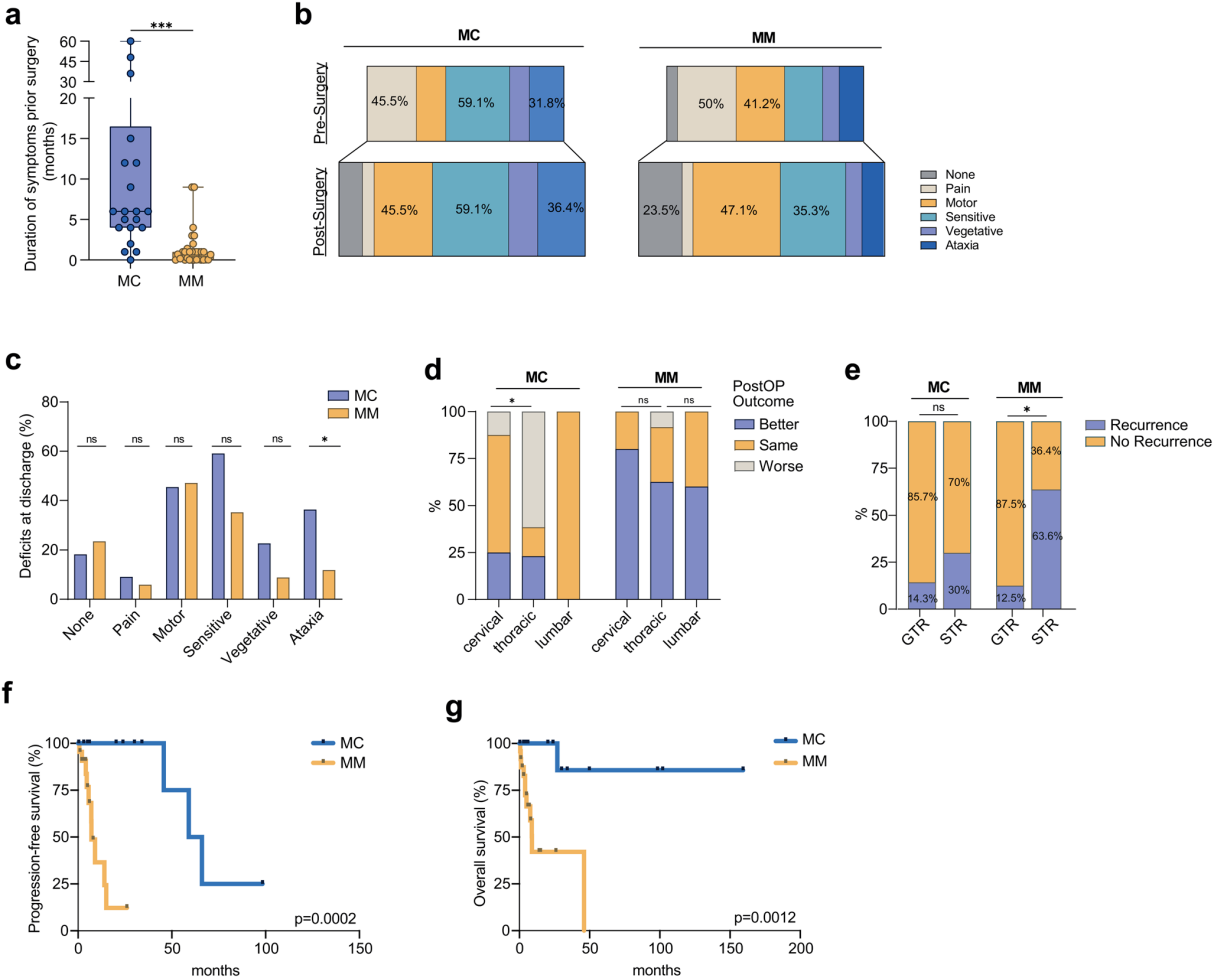
Neurological outcome and recurrence in spinal melanocytoma (MC) and malignant melanoma (MM). **a**) Duration of symptoms prior to surgery was significantly longer in MC than in MM (13.1 vs. 1.3 months, *p* = 0.0001). **b**) Pre- and postoperative neurological deficits stratified. *Data are presented as n (%). Percentages may exceed 100% due to multiple responses. **c**) Distribution of postoperative deficits at discharge, showing significantly higher rates of ataxia in MC compared to MM (36.4% vs. 11.8%, *p* = 0.0448). **d**) Postoperative outcome by spinal location: patients with MM showed significantly better improvement after surgery compared to MC (Better: 22.7% vs. 64.7%, *p* = 0.0012) and thoracic MC showed higher rates of deterioration compared to cervically located MCs. **e**) Recurrence rates stratified by extent of resection showed significantly fewer recurrences following GTR compared to STR in the MM group, whereas no statistically significant difference was observed in the MC cohort. **f**) Kaplan-Meier curves illustrating PFS between MC and MM patients with significantly longer PFS for MC compared to MM (7.1 vs. 62.5 months, HR 11.5, 95% CI 3.1–41.8, *p* = 0.0002) **g**) Kaplan-Meier curve illustrating OS differences between MC and MM. Patients with MC demonstrated significantly improved OS compared with MM (HR 7.2, 95% CI 2.2–23.9 *p* = 0.0012)

Symptom duration prior to surgery was significantly longer in MC (13.1 ± 15.9 months) than in MM (1.3 ± 2.2 months; *p* = 0.0001; Fig. 3a). Preoperative deficits were common in both groups, with a trend toward more sensory and ataxic symptoms in the MC group (Fig. 3b). However, ataxia at discharge was significantly more frequent in the MC group than in the MM group (36.4% vs. 11.8%; *p* = 0.0448; Fig. 3c).

Postoperative outcomes differed significantly within both groups, with 64.7% of MM patients showing neurological improvement, compared to only 22.7% in MC (*p* = 0.0012; Fig. 3d). Furthermore, we analyzed the influence of tumor location on surgical outcomes and found that thoracic MCs were associated with higher rates of postoperative deterioration than cervically located tumors. McCormick scores at discharge and the last follow-up, however, did not significantly differ between the groups.

Recurrence rates stratified by the EOR demonstrated significantly fewer recurrences in MM following GTR compared to STR (Fig. 3e), whereas no statistically significant difference was observed in the MC group. Local and distant recurrence rates were comparable across both cohorts. However, MM depicted significantly shorter progression-free survival compared to MCs (7.1 vs. 62.5 months, HR 11.5, 95% CI 3.1–41.8, *p* = 0.0002) (Fig. 3e). Adjuvant treatment was significantly more common in MM (*p* = 0.0001), with 44.1% of patients receiving combined RTx + CTx compared to 9.1% in MC. In MCs, adjuvant therapy was used in only a minority cases (n = 7/22, 31.8%), and postoperative RTx was applied in 5/22 patients (22.7%), primarily in situations where GTR could not be safely achieved (4/5, 80%). Follow-up was significantly longer in MC (36.3 ± 47.4 vs. 8.1 ± 9.8 months; *p* = 0.0072). Despite a shorter follow-up period, overall mortality was significantly higher in the MM group (41.2% vs. 4.5%; *p* = 0.0067) and MM showed a significantly shorter OS (HR 7.2, 95% CI 2.2–23.9 *p* = 0.0012) (Fig. 3f). To assess potential confounders, we performed a multivariable Cox proportional hazards analysis. Due to the limited number of events in this rare-disease cohort, a stable model could only be fitted for OS and had to be restricted to the most clinically relevant variables (histology, extent of resection, and adjuvant RTx). In this reduced model, histology remained the only independent predictor of OS, with malignant melanoma showing a significantly higher hazard of death compared with melanocytoma (HR 30.99, 95% CI 2.81-341.36, *p* = 0.005). Neither EOR (HR 0.90, 95% CI 0.31–2.62, *p* = 0.847) nor adjuvant RTx (HR 0.40, 95% CI 0.07–2.30, *p* = 0.305) reached statistical significance (Supplementary Table 2). No stable multivariable model could be generated for PFS due to the low number of events. In addition, we performed log-rank analysis to explore the effects of RTx in MCs. Here RTx was not significantly associated with OS (*p* = 0.5271, HR = 4.055, 95% CI 0.05–310.6) or PFS (*p* = 0.6949, HR = 1.153, 95% CI 0.06–6.32).

## Discussion

This multicenter retrospective study provides a comparative analysis of spinal intradural MCs and MM. While both tumors originate from melanocytic lineages, our findings highlight profound differences in their clinical, radiological, histopathological, and surgical characteristics, with important implications for diagnostic accuracy and therapeutic decision-making.

Macroscopic appearance of the lesion is a frequent challenge in the intraoperative management of melanocytic tumors. Due to their dark pigmentation and sometimes infiltrative growth, spinal MCs may visually resemble MMs and are radiographically hard to distinguish [8, 9, 14]. Surgeons encountering a jet-black tumor within the spinal cord or leptomeninges may instinctively conclude a diagnosis of metastatic melanoma, especially in the absence of a prior biopsy. This heuristic may be reinforced by the clinical observation that melanomas can remain clinically occult: the primary lesion may be atypically located, clinically silent, or may even have regressed spontaneously. Indeed, several case series of metastatic CNS melanoma describe patients without identifiable primary cutaneous lesions, complicating efforts to establish the origin of disease [10–12]. Consequently, pigmented spinal lesions without a known history of melanoma present a relevant diagnostic dilemma for the distinction between MM and metastatic melanoma to the spine with occult primary.

The current study provides evidence to inform such situations and aims to raise clinical awareness for the rare but distinct entity of spinal MC. Our data support the use of a more nuanced and evidence-based diagnostic approach when encountering melanotic spinal tumors. Specifically, we propose that, in the absence of a known melanoma diagnosis, surgeons and neuropathologists should consider the following four parameters: Ki-67 proliferation index, patient age, duration of preoperative symptoms, and tumor localization. In addition, Ki-67 may in principle also be assessed intraoperatively from cryo sections to support immediate decision-making in selected cases.

In our cohort, MCs were significantly more likely to occur in the craniocervical region and display an intramedullary growth pattern, whereas MMs were more broadly distributed along the spine and frequently demonstrated extraspinal extension. These anatomical patterns reflect fundamental differences in tumor biology: while MCs arise from meningeal melanocytes of the leptomeninges, MMs typically represent secondary seeding from extracranial disease. Of note, we observed that cervical MCs presented in younger patients, suggesting a potential age-related predilection for certain spinal segments. Moreover, a sex-specific age disparity was observed in MMs, with older male predominance, which may reflect differences in melanoma progression, immune surveillance, or detection biases. Histopathologically, the two tumor types diverged markedly in terms of proliferative activity. The Ki-67 index was significantly elevated in MMs, with cervical lesions showing particularly high proliferation. This index also emerged as a potential prognostic marker, with lower Ki-67 values in non-recurrent MMs, whereas MCs exhibited generally lower and more homogeneous Ki-67 levels. Interestingly, an inverse correlation between age and Ki-67 index was noted in MCs, suggesting that tumors presenting in younger patients may exhibit a relatively higher proliferative potential despite their histological classification as benign. Additionally, beyond conventional histopathology, extended molecular and DNA-methylation profiling may further aid in discriminating MCs from MMs, support risk stratification, and potentially inform individualized adjuvant treatment strategies. Additionally, beyond conventional histopathology, extended molecular and DNA-methylation profiling may further aid in discriminating MCs from MMs, support risk stratification, and potentially inform individualized adjuvant treatment strategies. In our cohort, molecular profiling was not performed systematically across all cases. Nonetheless, among MCs we identified one tumor harboring a GNAQ p.Q209L mutation and one case with an ATP2B4/PRKCA fusion. In line with previous reports, GNAQ and GNA11 mutations have been described in a substantial proportion of primary meningeal melanocytic tumors, with incidences of approximately 60–70% in published series [7, 15, 16]. BRAF status was assessed in 40.1% (*n* = 9) of all MC cases, with only one single case harboring a BRAF mutation, whereas MMs more frequently harbored BRAF-mutations (47.6%, *n* = 10/21 available cases). This is consistent to current literature that BRAF, HRAS, KRAS or KIT mutations occur rarely in meningeal MCs and are more commonly observed in cutaneous melanoma [7]. Taken together, these findings underscore the importance of integrating molecular profiling, both for diagnostic discrimination and for future studies assessing their impact on recurrence, survival, and suitability for targeted therapies.

Surgically, GTR was more frequently achieved in MMs, reflecting their often extramedullary localization and less adherent growth pattern. In contrast, MCs were more commonly intramedullary, posing substantial challenges to safe resection. Prior reports have emphasized the risk of neurological deficits during MC resection due to the lack of a clear cleavage plane and the tendency of these tumors to adhere tightly to surrounding neural structures [17, 18]. Consistent with these observations, postoperative deterioration—particularly ataxia—was more common in MCs, especially in thoracic locations, where spinal cord vascular anatomy is more complex and vulnerable.

Nonetheless, the overall functional outcome as measured by the McCormick scale showed no statistically significant differences between the two groups at discharge or follow-up, suggesting that despite early neurological deficits, some degree of recovery may be achieved over time with rehabilitation. Notably, stabilization procedures were exclusively performed in MM cases, underscoring their tendency for transdural growth, to invade vertebral structures and compromise spinal stability—features rarely observed in MC [17, 19–24].

The recurrence dynamics of both entities differed markedly. While MMs showed a clear benefit from GTR in terms of reduced recurrence rates, MCs did not exhibit a statistically significant correlation between EOR and recurrence. This may reflect the biological heterogeneity of MCs or the relatively small sample size. Importantly, this finding calls into question the necessity of aggressive surgical strategies in all cases of MC and supports the need for individualized treatment planning. Additionally, whereas adjuvant therapy was commonly employed in MMs — including immunotherapy, CTx, and RTx — it was infrequently used in MCs. In our cohort, adjuvant therapy was used in only a minority of MC cases (7/22, 31.8%), and adjuvant RT was applied in 5/22 patients (22.7%), primarily in situations where GTR could not be achieved. However, in univariate Kaplan-Meier testing, we did not discover any significant benefit in OS (*p* = 0.5271, HR = 4.055, 95% CI 0.05–310.6) nor in PFS (*p* = 0.6949, HR = 1.153, 95% CI 0.06–6.32) after RT in MCs. This aligns with current practice and supports the notion that MCs, although sometimes recurrent, do not necessarily require systemic therapy, particularly in the absence of malignant histological features or residual tumor growth. For MCs with STR or higher proliferative indices, adjuvant RTx may be considered on a case-by-case basis, as some studies have reported delayed local recurrence after RTx, although clear survival benefits remain unproven [25, 26]. In contrast, systemic therapy such as CTx or immunotherapy is not routinely recommended for MC and should be reserved for exceptional cases with atypically aggressive or recurrent behavior when surgery or RTx have been exhausted [4]. As apparently residual MC may remain silent or progress/recur for yet unknown reason, regular and long-term follow up over years is warranted in MC.

Mortality rates in our study reflect the systemic burden of metastatic melanoma [22, 23, 27]. Despite often aggressive local control, MM patients exhibited significantly higher mortality, typically due to extradural disease progression. In contrast, MC patients experienced significantly longer follow-up periods and markedly lower mortality, in line with their less aggressive behavior [20, 28].

The key translational message of this study is that not all “black spinal tumors” are the same. While the intraoperative visualization of a pigmented lesion may instinctively suggest malignant melanoma, the absence of a known primary, coupled with features such as a lower Ki-67 index, prolonged symptom duration, younger age, and intramedullary or craniocervical localization should prompt consideration of a spinal MC in the differential diagnosis. The implications are nontrivial: MCs do not always mandate adjuvant systemic therapy, and overly aggressive treatment based on incorrect assumptions may expose patients to unnecessary neurological risk.

In this context, our retrospective work provides a framework for more accurate intraoperative decision-making and postoperative care. We encourage surgeons and neuropathologists and to consider these parameters as decision aids, not determinants, and we advocate for greater awareness of spinal MC as a distinct pathological entity. As molecular profiling and immunohistochemical tools become more widely available, future studies should aim to define additional biomarkers that may further refine the differential diagnosis and guide therapeutic strategies.

In conclusion, this study underscores that melanocytic tumors of the spine, while visually similar, encompass biologically and clinically distinct entities. Spinal MC represents a rare but important differential diagnosis that requires careful consideration. Through the identification of clinical and histopathological predictors, we aim to promote a more nuanced understanding of “black tumors” in the spine and to guide more tailored, evidence-based management strategies, while acknowledging the need for prospective validation of these findings.

## Limitations

This multicenter study is limited by its retrospective design, potential institutional bias in surgical approach and adjuvant therapy, and the relatively small sample size inherent to the rarity of these tumors. In particular, the subgroup of MC remains small, and several analyses, such as the association between EOR and adjuvant therapy and recurrence, are likely underpowered to detect moderate effect sizes and no adjustments for multiple comparisons were applied. Accordingly, conclusions regarding the prognostic relevance of resection extent must therefore be drawn cautiously. Outcomes may have been influenced by center-level differences in surgical technique, perioperative pathways, and adjuvant strategies. The lack of centralized histopathological review may have introduced interobserver variability, although all centers applied WHO-2021 criteria, making major misclassification unlikely. The retrospective design spanning 14 years cannot exclude the possibility of minor inconsistencies in data collection. Follow-up duration differed between groups, being longer in MC compared to MM, which may have introduced a slight bias in outcome assessment. Clinical and radiological follow-up intervals were not experimentally standardized but followed institutional routine protocols. Postoperative MRI was typically performed within 72 h after surgery to assess the EOR, followed by serial clinical and radiological follow-up every 6–12 months for benign lesions (melanocytoma) and every 3–6 months for malignant lesions (melanoma), or as clinically indicated. Post-therapy imaging after RT or systemic treatment was reviewed whenever available. Nonetheless, the multicenter design strengthens the generalizability of our findings and provides one of the most comprehensive comparative evaluations of spinal melanocytic tumors to date, offering a more robust assessment than previously published single-institution case series.

## Conclusion

Although spinal MC and MM may appear radiologically similar at initial presentation, the current study demonstrated that they differ substantially in terms of their localization, growth pattern, proliferative activity, and clinical behavior. While MCs often present with intramedullary lesions and longer symptom duration, they have a more favorable prognosis but pose greater surgical challenges due to their anatomical location. MMs, on the other hand, are characterized by aggressive growth, high proliferation indices, and systemic dissemination, yet often show better neurological function following surgery. Our study demonstrated that these differences are reflected not only in histopathological profiles, such as Ki-67 expression, but also in the surgical approach required and the postoperative functional outcomes. These findings underline the importance of distinguishing between both entities early in the diagnostic process and tailoring treatment strategies, considering tumor biology, anatomical considerations, and expected postoperative recovery.

## Supplementary Information

Below is the link to the electronic supplementary material.

Supplementary Material 1

Supplementary Material 2

Supplementary Material 3

## Data Availability

No datasets were generated or analysed during the current study.

## Abbreviations

CNS: Central Nervous System
CSF: Cerebrospinal Fluid
CTx: Chemotherapy
EOR: Extent of Resection
GTR: Gross Total Resection
IONM: Intraoperative Neuromonitoring
MC: Melanocytomas
MM: Malignant Melanoma
mMM: Metastatic Malignant Melanoma
MRI: Magnetic Resonance Imaging
OS: Overall survival
PFS: Progression-free survival
pMM: Primary Malignant Melanoma
PMN: Primary Melanocytic Neoplasms
RT: Radiotherapy
STR: Subtotal Resection
WHO: World Health Organization
