# Supplementary material for: Benign or aggressive? Understanding spinal melanocytomas in comparison to malignant melanoma
Source: J Neurooncol. 2025 Dec 1;176(1):102. doi: 10.1007/s11060-025-05350-0 (PMC12669298; doi:10.1007/s11060-025-05350-0)
Supplement: Supplementary file 1 — Supplementary Material 1 [file 11060_2025_5350_MOESM1_ESM.docx]

| **Supplementary Table 1** | | |  |  |
| --- | --- | --- | --- | --- |
|  |  | **pMM** | **mMM** | **p-value** |
| n (%) | | 7 (20.6) | 27 (79.4) |  |
| **Age** - Median (Range) | | 60 (44-86) | 58 (32-84) | 0.45 |
| **Sex** - (%) | |  |  |  |
|  | Female | 5 (71.4) | 12 (44.4) | 0.39 |
|  | Male | 2 (28.6) | 15 (55.6) |  |
|  |  |  |  |  |
| **EOR** | |  |  |  |
|  | GTR – n (%) | 6 (85.7) | 15 (55.6) | 0.22 |
| **Ki-67** - Mean (SD) | | 22.8 (19.2) | 27.7 (17.8) | 0.63 |
| **Median PFS** (months) | | 2.8 | 7.1 | 0.24 |
| **Median OS** (months) | | 1.2 | 8.9 | **0.02** |
