# Supplementary material for: Benign or aggressive? Understanding spinal melanocytomas in comparison to malignant melanoma
Source: J Neurooncol. 2025 Dec 1;176(1):102. doi: 10.1007/s11060-025-05350-0 (PMC12669298; doi:10.1007/s11060-025-05350-0)
Supplement: Supplementary file 2 — Supplementary Material 2 [file 11060_2025_5350_MOESM2_ESM.docx]

| **Supplementary Table 2** | |  |  |  |
| --- | --- | --- | --- | --- |
|  |  |  |  |  |
|  |  | **Hazard Ratio** | **95% CI** | **p-value** |
|  | **Histology** | 30.99 | 2.81-341.36 | 0.0005 |
|  | MM vs. MC |  |  |  |
|  | **EOR** | 0.90 | 0.31-2.62 | 0.847 |
|  | STR vs. GTR |  |  |  |
|  | **Adjuvant Radiotherapy** | 0.40 | 0.07-2.30 | 0.305 |
