# Supplementary material for: Benign or aggressive? Understanding spinal melanocytomas in comparison to malignant melanoma
Source: J Neurooncol. 2025 Dec 1;176(1):102. doi: 10.1007/s11060-025-05350-0 (PMC12669298; doi:10.1007/s11060-025-05350-0)
Supplement: Supplementary file 3 — Supplementary Material 3 [file 11060_2025_5350_MOESM3_ESM.docx]

| **Supplementary Table 3** | |  |  |  |
| --- | --- | --- | --- | --- |
|  |  | **IONM** | **no IONM** | **p-value** |
| Total - n | | 28 | 27 |  |
| Age [years] - mean | | 59.7 | 54.6 | 0.6366 |
| Sex - n (%) | |  |  | 0.508 |
|  | Male | 17 (60.7) | 14 (51.9) |  |
|  | Female | 11 (39.3) | 13 (48.1) |  |
| Histology | |  |  | 0.123 |
|  | MC | 14 (50) | 8 (29.6) |  |
|  | MM | 14 (50) | 19 (70.4) |  |
| Location | |  |  |  |
|  | Intramedullary | 6 (42.9) | 2 (11.8) | **0.032** |
|  |  |  |  | 0.135 |
|  | Cervical | 8 (28.6) | 4 (14.8) |  |
|  | Thoracic | 19 (67.9) | 18 (66.7) |  |
|  | Lumbar | 1 (3.5) | 5 (18.5) |  |
|  |  |  |  |  |
| EOR - n (%) | |  |  | 0.140 |
|  | GTR | 18 (64.3) | 12 (44.4) |  |
|  | STR | 10 (35.7) | 15 (55.6) |  |
| Outcome | |  |  |  |
|  | New Deficits | 13 (46.4) | 4 (14.8) | **0.011** |
|  | Better Outcome at discharge | 9 (32.1) | 17 (62.9) | 0.076 |
|  | Better Outcome at FUP | 7 (25) | 6 (22.2) | 0.782 |
